# Influence of complete administration of adjuvant chemotherapy cycles on overall and disease-free survival in locally advanced rectal cancer: post hoc analysis of a randomized, multicenter, non-inferiority, phase 3 trial

**DOI:** 10.1186/s12885-018-4309-6

**Published:** 2018-04-03

**Authors:** Flavius Sandra-Petrescu, Florian Herrle, Iris Burkholder, Peter Kienle, Ralf-Dieter Hofheinz

**Affiliations:** 1 Surgical Department, University Medical Centre Mannheim, Theodor-Kutzer-Ufer 1-3, 68167 Mannheim, Germany; 2 Department of Nursing and Health, University of Applied Sciences of the Saarland, Goebenstr. 40, D-66117 Saarbruecken, Germany; 3 Day Treatment Center (TTZ), Interdisciplinary Tumor Center Mannheim (ITM) & III Medical Clinic, Theodor-Kutzer-Ufer 1-3, 68167 Mannheim, Germany

**Keywords:** Completeness of chemotherapy, Rectal cancer, Overall survival, Disease-free survival, Curative resection

## Abstract

**Background:**

A randomized trial demonstrated that capecitabine is at least as effective as fluorouracil in the adjuvant treatment of patients with locally advanced rectal cancer. However, not all patients receive all planned cycles of chemotherapy. Therefore it is of interest how complete or partial administration of chemotherapy influences oncological outcome.

**Methods:**

A post hoc analysis of a trial with 401 randomized patients, nine being excluded because of missing data, was performed. 392 patients (197 - capecitabine, 195 - fluorouracil) could be analyzed regarding the number of administered adjuvant chemotherapy cycles. In the subgroup of 361 patients with an overall survival of at least six months, five-year overall and disease-free survival were analyzed in respect to completion (complete vs. incomplete) of chemotherapy cycles. Survival rates and curves were calculated and compared using the log-rank test. The effect of completion of chemotherapy was adjusted for relevant confounding factors.

**Results:**

Two hundred fifty-one (64.0%) of analyzed patients received all postoperative scheduled cycles. Five-year overall survival was significantly better in these patients compared to the incomplete group (76.0 vs. 60.6%, *p* < 0.0001). Of 361 patients with an overall survival of at least six months, 251(69.5%) patients received all cycles. Five-year overall survival was also significantly better than in the incomplete group (76.0 vs. 66.4%, *p* = 0.0073). Five-year disease free survival was numerically better (64.9 vs. 58.7%, *p* = 0.0646; HR [not all cycles vs. all cycles] = 1.42 95% CI: [0.98, 2.07]). Cox regression models show a non-significant better OS (*p* = 0.061) and DFS (*p* = 0.083), if chemotherapy cycles were administered completely.

**Conclusion:**

Complete administration of chemotherapy cycles was associated with improved five-year overall and disease-free survival in patients with locally advanced rectal cancer.

**Electronic supplementary material:**

The online version of this article (10.1186/s12885-018-4309-6) contains supplementary material, which is available to authorized users.

## Background

Multimodal treatment of locally advanced rectal cancer has continuously improved, resulting in better oncological outcome of affected patients. Current standard includes optimized surgery as defined by low anterior resection and total mesorectal excision (LAR and TME), combined with neoadjuvant (chemo)radiotherapy (long-term or short-course radiotherapy) and adjuvant chemotherapy. Locoregional recurrences have thus been considerably reduced but distant metastases are still the main problem. Several modifications of the standard, individual- or risk-adapted, are currently being investigated in order to further improve prognosis in rectal cancer. Optimized and patient-tailored neoadjuvant chemoradiotherapy (CRT) concepts with the aim of reducing local and distant recurrences are currently being tested in clinical trials [1]. The use of magnetic resonance imaging (MRI) predicting the circumferential resection margin (CRM) is now widely used in order to avoid neoadjuvant CRT in low risk cases [2, 3]. Also the role of adjuvant chemotherapy in patients with locally advanced rectal cancer undergoing neoadjuvant CRT and TME is at this time a matter of debate [4]. Moreover, omission of both, surgery and adjuvant chemotherapy is evaluated in selected patients with a complete response achieved with neoadjuvant CRT [5].

However, according to German and other international guidelines, neoadjuvant CRT and fluorouracil based adjuvant chemotherapy remain a standard of care in the multimodal treatment of stage II – III rectal cancers (cT3–4 N0 M0, cT_any_ N1–2 M0:T1 – tumour invades submucosa, T2 – tumour invades muscularis propria, T3 – tumour invades trough the muscularis propria into pericolorectal tissues, T4 – tumour directly invades other organs or structures and/or perforates visceral peritoneum, N0 – no regional lymph node metastasis, N1 – metastasis in 1 to 3 regional lymph nodes, N2 – metastasis in 4 or more regional lymph nodes, M0 – no distant metastasis; c – clinical, p – pathologic; as defined by the AJCC-7ed) [6–8]. As capecitabine, as an adjuvant treatment regime, was shown to be non-inferior to 5-FU regarding relapse-free survival in stage III (cT_any_ N1–2 M0) colon cancer it was also investigated as perioperative treatment in locally advanced rectal cancer [8–10]. A phase 3 multicenter trial demonstrated that capecitabine was at least as effective as fluorouracil in the neoadjuvant CRT and adjuvant setting within a multimodal treatment concept for patients with stage II-III (c or pT3–4 N_any_, or c or pT_any_ N_positive_, M0) rectal cancer [8]. Overall survival (OS) was non-inferior in the capecitabine compared with the fluorouracil group. Interestingly disease-free survival (DFS) was proven to be better in the capecitabine group due to fewer distant metastases [10]. Therefore, adjuvant treatment obviously plays a role in controlling distant metastases and in this context it seems sensible to administer all planned adjuvant chemotherapy cycles in order to potentially maximize the oncological benefit. But not all patients indeed receive all planed chemotherapy cycles and there is scarce data on whether completeness of chemotherapy cycles (CoC) really has a relevant effect on oncological outcome and if so how large this effect is. In order to determine whether CoC influences five-year OS and DFS a post hoc re–analysis of the data from our previous phase-III study was performed [10].

## Methods

A detailed description of the trial including inclusion and exclusion criteria as well as randomization, statistical aspects and other eligibility criteria was previously given [10]. Shortly this was a two-arm, two-cohort, multicentre, randomized, open-label, non-inferiority, phase 3 trial comparing fluorouracil with capecitabine for perioperative treatment of patients with locally advanced rectal cancer. Initially designed as an adjuvant trial the protocol was amended to allow a neoadjuvant cohort. The trial protocol was approved by the institutional review boards of all participating centres. All participants provided written informed consent. Newly diagnosed patients with locally advanced rectal adenocarcinoma, stage II – III (pT3–4 N_any_ or pT_any_ N_positive_, M0 for adjuvant cohort respectively cT3–4 N_any_ or cT_any_ N_positive_, M0 for neoadjuvant cohort), with a distal tumour border < 16 cm from anal verge were recruited in 35 German institutions between March, 2002, and December, 2007 [8]. All patients were resected in curative intent as confirmed by the histological results (R0). A partial or total mesorectal excision (PME or TME) was performed for tumours localized in the upper third and lower two-thirds of rectum, respectively.

Patients were randomized into two groups, capecitabine or fluorouracil, in a 1:1 ratio using permuted blocks, with stratification by centre and pathological tumour stage. In the adjuvant cohort the capecitabine group was scheduled to receive two cycles of capecitabine (2500 mg/m^2^ days 1–14, repeated day 22), followed by CRT (50.4 Gy plus capecitabine 1650 mg/m^2^ days 1–38), then three cycles of capecitabine. Patients randomized into the neoadjuvant cohort were planned to receive CRT (50.4 Gy plus capecitabine 1650 mg/m^2^ daily) followed by radical surgery and afterwards five cycles of capecitabine (2500 mg/m^2^ per day for 14 days).

In the adjuvant cohort the planned therapy for patients receiving fluorouracil included two cycles of bolus fluorouracil (500 mg/m^2^ days 1–5, repeated day 29), followed by CRT (50.4 Gy plus infusional fluorouracil 225 mg/m^2^ daily), then two cycles of bolus fluorouracil. Patients in the neoadjuvant cohort received CRT (50.4 Gy plus infusional fluorouracil 1000 mg/m^2^ days 1–5 and 29–33) followed by radical surgery and afterwards four cycles of bolus fluorouracil (500 mg/m^2^ for 5 days).

The overall population of the study consisted of 392 patients. A further subgroup of 361 patients with an overall survival time of at least 6 months was analyzed. Comparisons were performed between patients receiving all (complete [CoC] - group) scheduled therapy cycles (5-FU arm: 5 cycles, capecitabine arm: 6 cycles) versus all patients receiving not all scheduled cycles (incomplete [non-CoC] - group = at least one cycle less of the planned chemotherapy).

### Statistical methods

Baseline characteristics were analyzed using descriptive statistics. OS was calculated from the date of randomization to the date of death. DFS was calculated from the date of randomization to the date of disease recurrence (metastasis or local recurrence), development of a second primary cancer (including non-colorectal carcinoma), or death from any other cause, whichever occurred first. OS as well as DFS were analyzed using censored failure times with the Kaplan-Meier method. Kaplan-Meier curves for OS and DFS were compared using the log-rank test. Five-year survival rates as well as Cox proportional hazard rates (HR) and 95% confidence intervals were calculated for OS and DFS. In addition, univariate Cox regression analysis was performed for completion of chemotherapy as well as for all baseline characteristics of the study population (age, gender, therapy arm, cohort, WHO status, tumor and nodal category). Cox proportional HRs as well as 95% confidence intervals were calculated. Global null hypothesis was tested using likelihood ratio test. Parameters which were significant in univariate analysis at a 5% significance level were included in a multivariate Cox regression with the aim to adjust the effect of completion of chemotherapy for relevant confounding factors. Univariate as well as multivariate Cox regression was performed for OS and DFS. Data were analyzed using the statistical package SAS for Windows version 9.4 (SAS Institute Inc., North Carolina).

## Results

**Fig. 1:**
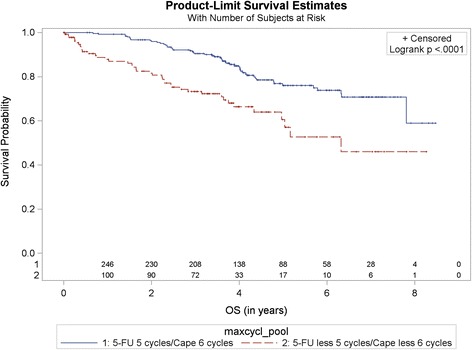
Kaplan-Meier curve for overall survival (in years). Comparison by treatment and number of cycles. Pooled analysis of 5-FU vs. capecitabine, CoC (5-FU 5 cycles/capecitabine 6 cycles) vs. non-CoC (5-FU less 5 cycles/capecitabine less 6 cycles). Cape – capecitabine. 1and 2 – number at risk

Of 401 randomized patients nine were excluded because of missing post-randomization data. Therefore 392 patients were analyzed, 197 in the capecitabine and 195 in the fluorouracil group. Thereof, 231 patients were included in the adjuvant cohort and 161 in the neoadjuvant cohort. 251 (64%) patients in both groups, capecitabine and fluorouracil, respectively completed the planned adjuvant chemotherapy cycles (6 cycles capecitabine and 5 cycles 5-FU). CoC group (*N* = 251) and non-CoC group (*N* = 141) were compared by treatment and number of cycles. The pooled analysis of 5-FU and capecitabine CoC vs. non-CoC cycles showed a significantly better 5-year OS 76% (95% CI 69.1–81.6%) in the CoC group vs. 60.6% (95% CI 48.0–71.0%, *p* < 0.0001) in the non-CoC group (Fig. 1).

A total of *N* = 31 patients of the original population have OS lower than 6 months. Reasons for end of study of those patients are:

- Death (10 patients- among them 2 tumour related, 1 therapy related, 3 unrelated to tumour or therapy, 4 missing reasons)
- Withdrawal of consent (11 patients)
- Lost to follow-up (1 patient)
- Protocol deviation (3 patients)
- Toxicity (1 patient)
- Death and toxicity (1 patient)
- Others (4 patients).

Looking further at the subgroup of patients with at least 6 months OS (Table 1), 251 (69.53%) of 361 patients, received all cycles (CoC) and 110 (30.47%) patients received at least one cycle less (non-CoC) (Table 2). Most patients have a T3 tumour (clinical or pathological) and are node positive.

**Table 1:** Baseline characteristics of the study population: patients with at least 6 months OS (*N* = 361)

	CoC-Group (*N* = 251)	non-CoC-Group (*N* = 110)
Median age, years (range; interquartile range)	63 (30–83; 56.1–68.7)	65 (40–86; 56.4–71.5)
Sex		
Male	164 (65%)	77 (70%)
Female	87 (35%)	33 (30%)
WHO status		
0	139 (55%)	63 (57%)
1	90 (36%)	40 (36%)
2	2 (1%)	2 (2%)
Missing data	20 (8%)	5 (5%)
Therapy arm		
Capecitabine	127 (51%)	53 (48%)
5-FU	124 (49%)	57 (52%)
Cohort		
Adjuvant	182 (73%)	36 (33%)
Neoadjuvant	69 (27%)	74 (67%)
Tumour category^a^		
T1 or T2	48 (19%)	12 (11%)
T3	184 (73%)	89 (81%)
T4	18 (7%)	8 (7%)
Missing data	1 (< 1%)	1 (1%)
Nodal category^a^		
Node negative	93 (37%)	45 (41%)
Node positive	157 (63%)	60 (54%)
Missing data	1 (< 1%)	5 (5%)

Data are n (%) or median (range). ^a^clinical or pathological category. *WHO* World Health Organization, *FU* fluorouracil. T - size or direct extent of the primary tumor

**Table 2:** Patients receiving scheduled treatment per cycle

	Capecitabine	Fluorouracil
Adjuvant Cohort^1^		
1	107 (98%)	109 (100%)
2	105 (96%)	106 (97%)
3	102 (94%)	101 (93%)
4	99 (91%)	96 (88%)
5	98 (90%)	92 (84%)
6	90 (83%)	–
Neoadjuvant Cohort^2^		
1	66 (93%)	71 (99%)
2	50 (70%)	46 (64%)
3	46 (65%)	38 (53%)
4	42 (59%)	35 (49%)
5	40 (56%)	32 (44%)
6	37 (52%)	–

Data are n (%)

^1^*n* = 109 Capecitabine, *n* = 109 Fluorouracil;

^2^*n* = 71 Capecitabine, *n* = 72 Fluorouracil

Looking further at the number of received cycles, 12 (33%) of 36 patients (8 capecitabine and 4 5-FU) in the adjuvant cohort and 9 (12%) of 74 patients (3 capecitabine and 3 5-FU) in the neoadjuvant cohort received one cycle less than planned (Table 2). Overall, 19% of patients in the non-CoC group received only one cycle less. Looking at the chemotherapy regimens within the non-CoC group, 11 (10%) patients in the capecitabine group and 7 (6%) patients in the 5-FU group received one cycle less than the planned therapy.

**Fig. 2:**
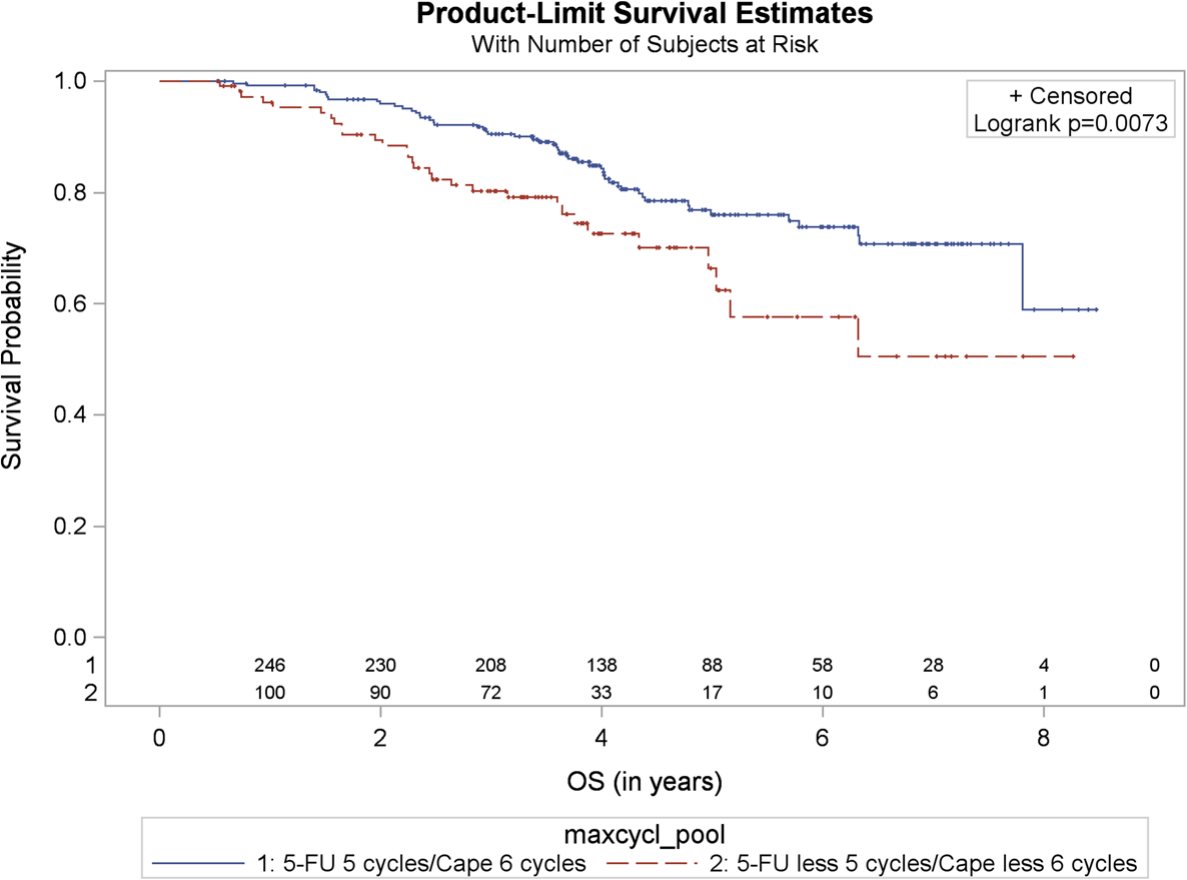
Kaplan-Meier curve for overall survival in patients with at least 6 months survival (in years). Comparison by treatment and number of cycles. Pooled analysis of 5-FU vs. capecitabine, CoC (5-FU 5 cycles/capecitabine 6 cycles) vs. non-CoC (5-FU less 5 cycles/capecitabine less 6 cycles). Cape – capecitabine. 1 and 2 – number at risk

The analysis of this subgroup of population showed a significantly better OS in the CoC group compared to the non-CoC group, 76.0% (95% CI 69.1%, 81.6%) vs. 66.4% (52.7%, 76.9%), *p* = 0.0073; HR for non-CoC vs. CoC 1.84 95% CI: [1.17, 2.90] (Fig. 2).

**Fig. 3:**
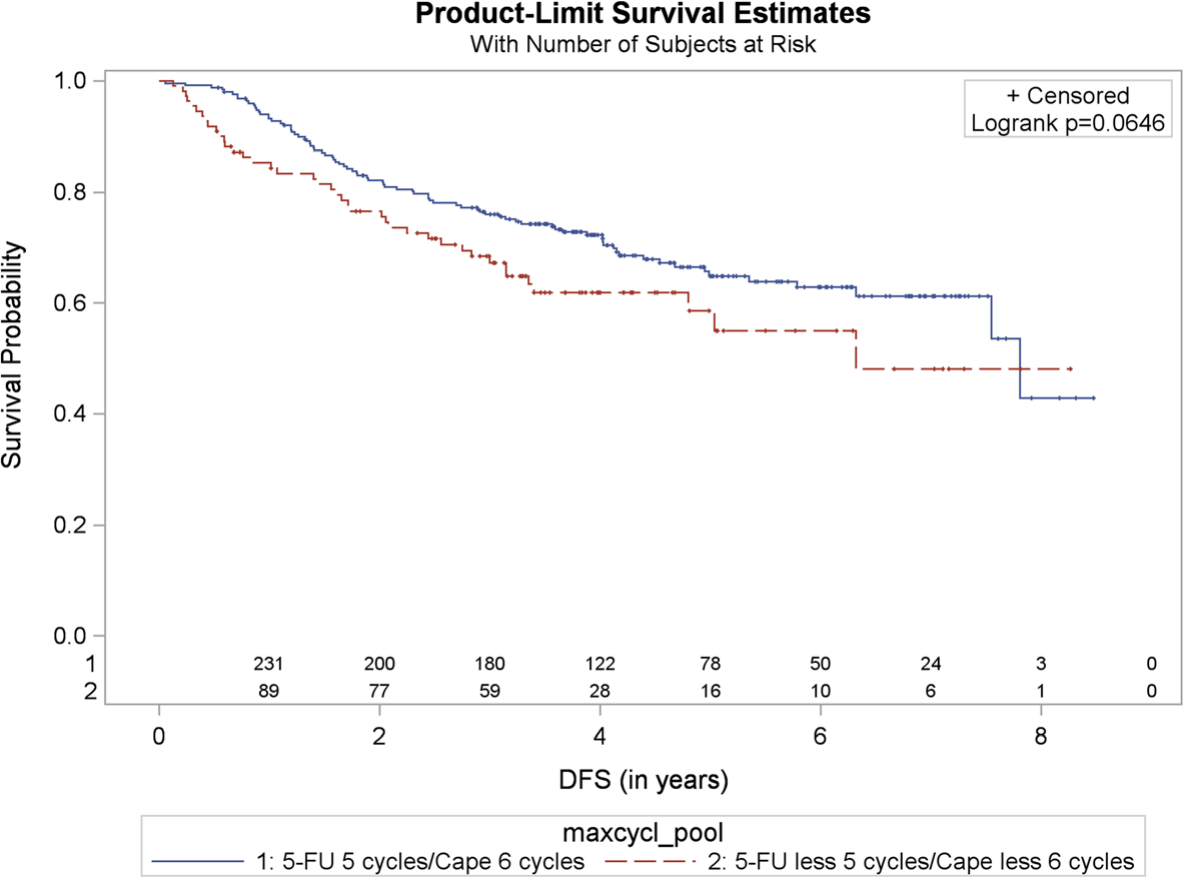
Kaplan-Meier curve for disease free survival in patients with at least 6 months survival (in years). Comparison by treatment and number of cycles. Pooled analysis of 5-FU vs. capecitabine, CoC (5-FU 5 cycles/capecitabine 6 cycles) vs. non-CoC (5-FU less 5 cycles/capecitabine less 6 cycles). Cape – capecitabine. 1and 2 – number at risk

The pooled analysis of 5-year disease-free survival showed a non-significant better outcome in the CoC group 64.9% (95% CI 57.8–71.0%) compared to the non-CoC group 58.7% (95% CI46.7–68.8%, *p* = 0.0646); HR for non-CoC vs. CoC was 1.42 (95% CI 0.98–2.07) (Fig. 3). Univariate as well as multivariate Cox regression analyses of OS and DFS by baseline characteristics for the study population are presented as Additional file 1.

## Discussions

In our post hoc analysis complete administration of planned adjuvant chemotherapy (6 cycles capecitabine and 5 cycles 5-FU) led to a better survival in patients with locally advanced rectal cancer. The Cox regression shows that even after adjustment CoC still leads to a clinically relevant, yet not statistically significant better survival. When looking specifically at the different chemotherapy regimens but also at the adjuvant and neoadjuvant cohort most of the patients within the non-CoC group received less than only one cycle less than complete and this resulted in a worse oncological outcome. These results and others indicate that it is of importance that patients undergo all chemotherapy cycles in order to maximally benefit from adjuvant treatment [11]. A large randomized controlled trial comparing neoadjuvant to adjuvant treatment in rectal cancer demonstrated CoC in only 50.0% of patients [12]. This is similar to a CoC rate of 57.0% in a randomized study comparing induction chemotherapy prior to neoadjuvant chemo-radiotherapy [11]. In our study CoC pooled for both cohorts, adjuvant and neoadjuvant, capecitabine and 5-FU, was 64.0% if the entire population of the study was considered (*N* = 392) or 69.5% in case of the subgroup of patients with at least 6 months survival (*N* = 361). This is comparable to a recent retrospective analysis of 294 patients with rectal cancer, stage I-IV (IV – T_any_ N_any_ M1: M1 – distant metastasis), undergoing neoadjuvant treatment, rectal resection with protective ileostomy and adjuvant therapy [8, 13]. In this study 65% of the patients received a complete adjuvant therapy, which in contrast to our trial, was broadly defined as application of complete adjuvant chemotherapy for at least 3 months [13]. The multimodal therapy of locally advanced rectal cancer is continuously improving and neoadjuvant as well as adjuvant therapy strategies are currently, in the era of optimized surgery (TME), under discussion. Regarding neoadjuvant CRT several patient orientated concepts are developing [1]. These include organ preserving strategies, non–operative management, induction or consolidation chemotherapy and use of targeted agents [1, 5]. The German and also most of the international guidelines still recommend adjuvant therapy of stage II – III rectal cancers, subsequent to neoadjuvant long course CRT or short-term radiotherapy (5 × 5 Gy) and TME [6, 8, 14]. Contrarily, a recently published meta-analysis of four clinical trials concluded that adjuvant fluorouracil-based chemotherapy does not improve OS or DSF in rectal cancer patients with (y)pTNM stage II-III disease [4, 8]. These patients received adjuvant chemotherapy or underwent observation after (chemo)radiotherapy and surgery. We and others already commented the results of this analysis as the included studies all have considerable drawbacks in regard to their validity [15–17]. In the meta-analysis no data is provided regarding the influence of CoC and of dose intensity on overall survival which was the primary endpoint of the analysis. As also stated by the authors, there was poor compliance to adjuvant therapy since less than 50% of the patients received the planned dose [4, 15–18]. No comparison between CoC and non-CoC was performed. This would be interesting since a complete (dose and cycles) administration of the chemotherapy might influence survival as now shown in our post hoc analysis. You et al. pointed out that it would be necessary, in order to analyze the effect of adjuvant chemotherapy on OS, to compare OS of patients who completely or incompletely received the adjuvant chemotherapy versus that of the observational group [16]. Patient selection in the studies included in the meta-analysis is somewhat unclear as a substantial number of patients from the I-CNR-RT and EORTC 2291 or those with an ypTNM0 and ypTNM1 tumour were excluded [8, 19, 20]. Moreover, a representative number of patients included into the analysis were from the PROCTOR-SCRIPT study which was closed prematurely and where a potential selection bias is evident [15]. Also patients with non-standard treatments such as 45Gy radiation without chemotherapy were included. Petrelli et al. argues that adjuvant chemotherapy should be especially considered in patients with locally advanced disease who experienced downstaging after neoadjuvant (chemo)radiotherapy [18]. But the patients included into the above cited meta-analysis were most probably mostly non-responders to neoadjuvant (chemo)radiotherapy since no or only minimally downstaging was achieved in this study.

In a pooled analysis of 11 studies Maas et al. showed that adjuvant therapy in rectal cancer may possibly be stratified on the basis of pathologic complete response (pCR) [21]. The authors found that after neoadjuvant CRT and TME ypT1–2 tumors benefited more from adjuvant treatment than ypT3–4 tumors [8, 21]. But also in this study the limited effect of chemotherapy is potentially due to poor adherence to adjuvant therapy.

There is evidence that different combinations of chemotherapy regimens may be more suitable in selected patient groups. Therefore, recent clinical studies investigated whether addition of oxaliplatin to the bolus fluorouracil and capecitabine is more effective in the perioperative treatment of locally advanced rectal cancer. In several of these studies (e.g. PETACC-6) neoadjuvant CRT showed high toxicity in the experimental arm (addition of oxaliplatin to standard capecitabine- or 5-FU-based regimen) [22]. This resulted in less compliance in the PETACC-6 experimental group when compared to the standard arm (approximately 77% of the patients started adjuvant chemotherapy, less in the oxaliplatin group and 69% completed it in the standard capecitabine arm and only 57% in the oxaliplatin arm). In the CAO/ARO/AIO-04 study, grade 3–4 side-effects were slightly more common in the 5-FU/oxaliplatin combination group (21 vs. 15%), but this had no effect on the rate of applied full chemotherapy dose [23]. In the PETACC-6 study, the primary endpoint was not reached (DFS standard arm 74.5% and + oxaliplatin 73.9%, *p* = 0.78) whereas in the CAO/ARO/AIO-04 study there was a significant improvement of 3-year-DFS in the 5-FU + oxaliplatin group (75.9 vs. 71.2%, *p* = 0.03).

The Korean phase II study ADORE evaluated whether addition of oxaliplatin to adjuvant therapy is of advantage in “high risk” histology [24]. 95% of patients completed planned chemotherapy cycles and 3-year-DFS was significantly better in patients receiving oxaliplatin (72 vs. 63%, *p* = 0.03), however restricted to patients with stage III disease [8, 24].

Although ADORE and CAO/ARO/AIO-04 trials showed a significant advantage of adjuvant chemotherapy regarding DFS when compared combined fluorouracil and oxaliplatin to fluorouracil alone, no further investigations were performed to determine whether complete vs. incomplete administration of the adjuvant chemotherapy significantly improve the OS or DFS [23, 24].

Our data show that CoC improves long-term oncological results, OS and DFS respectively, in rectal cancer patients when compared to incomplete adjuvant therapy. In our analysis considerably more patients underwent all chemotherapy cycles in the adjuvant compared to the neoadjuvant group and this was similar in the capecitabine and 5-FU group. This is might be explained due to the additional side-effects of concomitant radiotherapy in the latter group. A higher rate of gastrointestinal side-effects like proctitis and diarrhoea was noticed during radiotherapy in the capecitabine group [10]. Moreover, acute and chronic side-effects of neoadjuvant CRT or short course radiotherapy followed by surgery have been described to influence the postoperative course of the patients [25–29]. The toxicity of neoadjuvant therapy was shown to influence the rate of postoperative complications when compared to surgery alone [30]. A substantial percentage of patients will not be able to complete the scheduled adjuvant therapy. This is mostly due to suboptimal compliance with the therapy which can be explained by chemotherapy toxicity and postoperative complications such as stoma-related morbidity, high-output stoma, stoma prolapse or bowel occlusion, wound infection or anastomotic leakage [11, 31–34]. To improve CoC we designed a multicentre prospective randomized trial which addresses one of these factors which influence postoperative morbidity. This trial is analyzing the influence of a protective stoma on the administration of the complete adjuvant chemotherapy in patients with stage II – III rectal cancer [8, 35]. CoC is the primary endpoint and the trial will allow us also to evaluate the influence of dose reduction on patients’ survival. This trial is currently recruiting and 231 patients are randomized until now.

## Conclusions

Complete administration of scheduled adjuvant chemotherapy cycles was associated with improved survival in patients with locally advanced rectal cancer. Patient compliance should be improved in order to increase the rate of completeness of chemotherapy.

## Additional file

Additional file 1: Uni- and multivariate analyses for OS and DFS. Univariate Cox regression models for CoC and baseline characteristics of the study population as well as adjustment of CoC effect for relevant confounding factors. (DOCX 45 kb)

## Abbreviations

CoC: completeness of chemotherapy cycles
CRM: circumferential resection margin
CRT: chemoradiotherapy
DFS: disease - free survival
FU: fluorouracil
LAR: low anterior resection
MRI: magnetic resonance imaging
OS: overall survival
pCR: pathologic complete response
PME: partial mesorectal excision
TME: total mesorectal excision
Vs.: versus
