# Supplementary material for: Influence of complete administration of adjuvant chemotherapy cycles on overall and disease-free survival in locally advanced rectal cancer: post hoc analysis of a randomized, multicenter, non-inferiority, phase 3 trial
Source: BMC Cancer. 2018 Apr 3;18:369. doi: 10.1186/s12885-018-4309-6 (PMC5883296; doi:10.1186/s12885-018-4309-6)
Supplement: Supplementary file 1 — Uni- and multivariate analyses for OS and DFS. Univariate Cox regression models for CoC and baseline characteristics of the study population as well as adjustment of CoC effect for relevant confounding factors. (DOCX 45 kb) [file 12885_2018_4309_MOESM1_ESM.docx]

**Univariate and multivariate analyses for OS and DFS**

1. **Cox-Regression for OS**

**Table A1: Results for univariate Cox-Regression**

|  | n | events | HR | 95%-CI | p-value^2^ |
| --- | --- | --- | --- | --- | --- |
| **Completion**  **CoC**  **non-CoC** | 251 110 | 52 30 | Ref 1.842 | [1.171-2.898] | 0.011 |
| **Age (years)** | 361 | 82 | 1.021 | [0.996-1.046] | 0.097 |
| **Sex**  **Male**  **Female** | 241  120 | 57  25 | Ref  0.891 | [0.556-1.428] | 0.629 |
| **Therapy arm**  **Capecitabine 5-FU** | 180 181 | 51  31 | Ref  1.718 | [1.099-2.684] | 0.016 |
| **Cohort**  **Adjuvant**  **Neoadjuvant** | 218 143 | 48 34 | Ref 1.774 | [1.112-2.831] | 0.018 |
| **WHO status**  **0**  **1**  **2**  **Missing** | 202  130  4  25 | 43  32  1  6 | Ref  1.113  0.720  1.698 | [0.704-1.761]  [0.099-5.249]  [0.721-3.998] | 0.676 |
| **Tumour category^1^**  **T1 or T2**  **T3**  **T4**  **Missing** | 60  273  26  2 | 7  66  8  1 | Ref  2.126  3.111  7.025 | [0.975-4.635]  [1.127-8.589]  [0.860-57.359] | 0.073 |
| **Nodal category^1^**  **Node negative**  **Node positive**  **Missing** | 138  217  6 | 24  57  1 | Ref  1.566  1.264 | [0.972-2.524]  [0.170-9.366] | 0.166 |

^1^ clinical or pathological category
 ^2^ Likelihood ratio test for global null hypothesis

The effect of completion of chemotherapy was adjusted by the significant parameters of the univariate Cox-Regression, therapy arm and cohort.

**Table A2: Multivariate Cox-Regression for OS**

|  | HR | 95%-CI | p-value^1^ |
| --- | --- | --- | --- |
| **Completion**  **CoC**  **non-CoC** | Ref 1.591 | [0.978-2.588] | 0.061 |
| **Therapy arm**  **Capecitabine 5-FU** | Ref  1.681 | [1.075-2.628] | 0.023 |
| **Cohort**  **Adjuvant**  **Neoadjuvant** | Ref  1.484 | [0.902-2.443] | 0.120 |

^1^ Wald test

1. **Cox-Regression for DFS**

**Table A3: Univariate Cox-Regression for DFS**

|  | n | events | HR | 95%-CI | p-value^2^ |
| --- | --- | --- | --- | --- | --- |
| **Completion**  **CoC**  **non-CoC** | 251 110 | 83 41 | Ref 1.423 | [0.977-2.072] | 0.072 |
| **Age (years)** | 361 | 124 | 1.015 | [0.995-1.035] | 0.143 |
| **Sex**  **Male**  **Female** | 241  120 | 84  40 | Ref  0.960 | [0.658-1.399] | 0.829 |
| **Therapy arm**  **Capecitabine 5-FU** | 180 181 | 50  74 | Ref  1.568 | [1.095-2.245] | 0.013 |
| **Cohort**  **Adjuvant**  **Neoadjuvant** | 218 143 | 76 48 | Ref 1.258 | [0.866-1.826] | 0.232 |
| **WHO status**  **0**  **1**  **2**  **Missing** | 202  130  4  25 | 66  48  1  9 | Ref  1.117  0.606  1.401 | [0.769-1.622]  [0.084-4.385]  [0.697-2.815] | 0.713 |
| **Tumour category^1^**  **T1 or T2**  **T3**  **T4**  **Missing** | 60  273  26  2 | 15  97  11  1 | Ref  1.535  2.134  2.501 | [0.891-2.645]  [0.979-4.650]  [0.330-18.969] | 0.231 |
| **Nodal category^1^**  **Node negative**  **Node positive**  **Missing** | 138  217  6 | 33  88  3 | Ref  1.822  2.942 | [1.221-2.719]  [0.901-9.611] | 0.005 |

^1^ clinical or pathological category
^2^ Likelihood ratio test for global null hypothesis

The effect of completion of chemotherapy was adjusted by the significant parameters of the univariate Cox-Regression, therapy arm and nodal category.

**Table A4: Multivariate Cox-Regression for DFS**

|  | HR | 95%-CI | p-value^2^ |
| --- | --- | --- | --- |
| **Completion**  **CoC**  **non-CoC** | Ref 1.401 | [0.957-2.052] | 0.083 |
| **Therapy arm**  **Capecitabine 5-FU** | Ref  1.534 | [1.070-2.198] | 0.020 |
| **Nodal category^1^**  **Node negative**  **Node positive**  **Missing** | Ref  1.809  2.655 | [1.212-2.701] [0.802-8.797] | 0.004  0.110 |

^1^ clinical or pathological category
^2^ Wald test
